# Prevalence and risk of developing sexual dysfunction in women with multiple sclerosis (MS): a systematic review and meta-analysis

**DOI:** 10.1186/s12905-023-02501-1

**Published:** 2023-07-04

**Authors:** Amid Yazdani, Narges Ebrahimi, Omid Mirmosayyeb, Mahsa Ghajarzadeh

**Affiliations:** 1 Department of Physical Medicine and Rehabilitation, Isfahan University of Medical Sciences, Isfahan, Iran; 2 Isfahan Neurosciences Research Center, Isfahan University of Medical Sciences, Isfahan, Iran; 3 Universal Council of Epidemiology (UCE), Universal Scientific Education and Research Network (USERN), Tehran University of Medical Sciences, Tehran, Iran

**Keywords:** Sexual dysfunction, Multiple sclerosis, Prevalence

## Abstract

**Objective:**

To estimate the pooled prevalence of sexual dysfunction (SD) in women with multiple sclerosis (MS).

**Methods:**

We systematically searched PubMed, Scopus, EMBASE, Web of Science, and google scholar and also gray literature up to October 2021.

The search strategy includes:

(“Multiple Sclerosis” OR “MS” OR “Disseminated Sclerosis” OR (Disseminated AND Sclerosis) OR (Sclerosis AND Multiple)) AND (“Sexual Dysfunction” OR (Sexual AND Dysfunction) OR (Sexual AND Dysfunctions) OR (Sexual AND Disorders) OR (Sexual AND Disorder) OR “Sexual Dysfunctions” OR “Sexual Disorders” OR “Sexual Disorder” OR “Psychosexual Dysfunctions” OR (Dysfunction AND Psychosexual) OR (Dysfunctions AND Psychosexual) OR “Psychosexual Dysfunction” OR “Psychosexual Disorders” OR (Disorder AND Psychosexual) OR (Disorders AND Psychosexual) OR “Psychosexual Disorder” OR “Hypoactive Sexual Desire Disorder” OR “Sexual Aversion Disorder” OR (Aversion Disorders AND Sexual) OR (Disorders AND Sexual Aversion) OR “Sexual Aversion Disorders” OR “Orgasmic Disorder” OR (Disorders AND Orgasmic) OR “Orgasmic Disorders” OR “Sexual Arousal Disorder” OR (Arousal Disorders AND Sexual) OR (Disorders AND Sexual Arousal) OR “Sexual Arousal Disorders” OR “Frigidity”).

**Results:**

We found 2150 articles by literature search, after deleting duplicates 1760 remained. Fifty-six articles remained for meta-analysis. The pooled prevalence of SD in MS patients estimated as 61% (95%CI:56–67%) (I^2^:95.7%, *P* < 0.001). The pooled prevalence of Anorgasmia in MS patients estimated as 29% (95%CI:20–39%) (I^2^:85.3%, *P* < 0.001). The pooled odds of developing SD in MS women estimated as 3.05(95%CI: 1.74–5.35) (I^2^:78.3%, *P* < 0.001). The pooled prevalence of decreased vaginal lubrication in MS patients estimated as 32%(95%CI:27–37%) (I^2^ = 94.2%, *P* < 0.001). The pooled prevalence of reduced libido was 48%(95%CI:36–61%) (I^2^:92.6%, *P* < 0.001). The pooled prevalence of arousal problems was 40%(95%CI: 26–54%) (I^2^:97.4%, *P* < 0.001). The pooled prevalence of intercourse satisfaction was 27% (95%CI: 8–46%) (I^2^:99%, *P* < 0.001).

**Conclusion:**

The result of this systematic review and meta-analysis show that the pooled prevalence of SD in women with MS is 61% and the odds of developing SD in comparison with controls is 3.05.

## Introduction

Multiple sclerosis (MS) is a degenerative, neurologic disease of the central nervous system (CNS) affecting women more than men [1]. It usually occurs between 20 and 50 years of age, while MS-related complications include both physical and psychological consequences [2]. One of the most common multi-dimensional complications is sexual dysfunction (SD) involving physiological, psychosocial, and interpersonal factors [3]. It is suggested that women with MS have problems regarding finding a partner, building a relationship, and marital issues [3]. SD has negative impacts on health-related quality of life (HRQoL), especially on youth [4, 5]. It can be found at any stages of the disease, and is present at early stage in some cases [6, 7]. The exact etiology of SD in MS is not clear, but physical disability, psychological difficulties, and also side effects of medications could cause SD [8, 9].

Primary SD is the consequence of neurological changes in the body, while secondary SD is due to MS-related complications such as fatigue, pain, spasticity, bladder, and bowel dysfunction [10]. Tertiary SD is related to psychological consequences of MS such as depression, anxiety, and cognitive impairment / and cultural issues regarding sexual consultant in different nations [10].

Loss of orgasm, libido, lubrication, and increased spasticity are common during sexual activity in women with MS  [11, 12].

Different factors such as age, disease duration, disability level, bladder dysfunction, cognitive impairment, and disease course influence SD in MS women [13–15].

Up to now, different original studies have been conducted and three previous systematic reviews and meta-analyses estimated the pooled prevalence of SD in women with MS [16–18]. The aim of this system and meta-analysis is to update the prevalence of SD in MS women.

## Methods

### Eligibility criteria

#### Inclusion criteria were

Cross-sectional studies, Articles that had been published in the English language.

#### Exclusion criteria

Case-report, RCT studies.

We followed The Preferred Reporting Items for Systematic reviews and Meta-Analyses (PRISMA) 2020 for reporting this systematic review [19].

### Information sources

Two independent researchers systematically searched PubMed, Scopus, EMBASE, Web of Science, and google scholar and also gray literature up to October 2021.

### The search strategy includes

(“Multiple Sclerosis” OR “MS” OR “Disseminated Sclerosis” OR (Disseminated AND Sclerosis) OR (Sclerosis AND Multiple)) AND (“Sexual Dysfunction” OR (Sexual AND Dysfunction) OR (Sexual AND Dysfunctions) OR (Sexual AND Disorders) OR (Sexual AND Disorder) OR “Sexual Dysfunctions” OR “Sexual Disorders” OR “Sexual Disorder” OR “Psychosexual Dysfunctions” OR (Dysfunction AND Psychosexual) OR (Dysfunctions AND Psychosexual) OR “Psychosexual Dysfunction” OR “Psychosexual Disorders” OR (Disorder AND Psychosexual) OR (Disorders AND Psychosexual) OR “Psychosexual Disorder” OR “Hypoactive Sexual Desire Disorder” OR “Sexual Aversion Disorder” OR (Aversion Disorders AND Sexual) OR (Disorders AND Sexual Aversion) OR “Sexual Aversion Disorders” OR “Orgasmic Disorder” OR (Disorders AND Orgasmic) OR “Orgasmic Disorders” OR “Sexual Arousal Disorder” OR (Arousal Disorders AND Sexual) OR (Disorders AND Sexual Arousal) OR “Sexual Arousal Disorders” OR “Frigidity”).

### Selection process

After obtaining the results, and importing them to Endnote, they omitted duplicates. Then titles, and abstracts were screening, and potential full texts were obtained. The researchers extracted data from each study, entered in Excel, and in the case of discrepancies, the third researcher solved the problem.

### Data items

Data regarding first author, country of origin, number of enrolled patients, number of cases with SD, mean age, mean EDSS, mean duration of the disease, were collected.

### Statistical analysis

All statistical analyses were performed using STATA (Version 14.0; Stata Corp LP, College Station, TX, USA). To determine heterogeneity, Inconsistency (I^2^) was calculated.

We used random effects model.

### Effect measures

The pooled prevalence of domains of sexual function were estimated. The pooled odds ratio(OR) of developing sexual dysfunction in women with MS comparing to healthy controls were calculated.

## Results

**Fig. 1:**
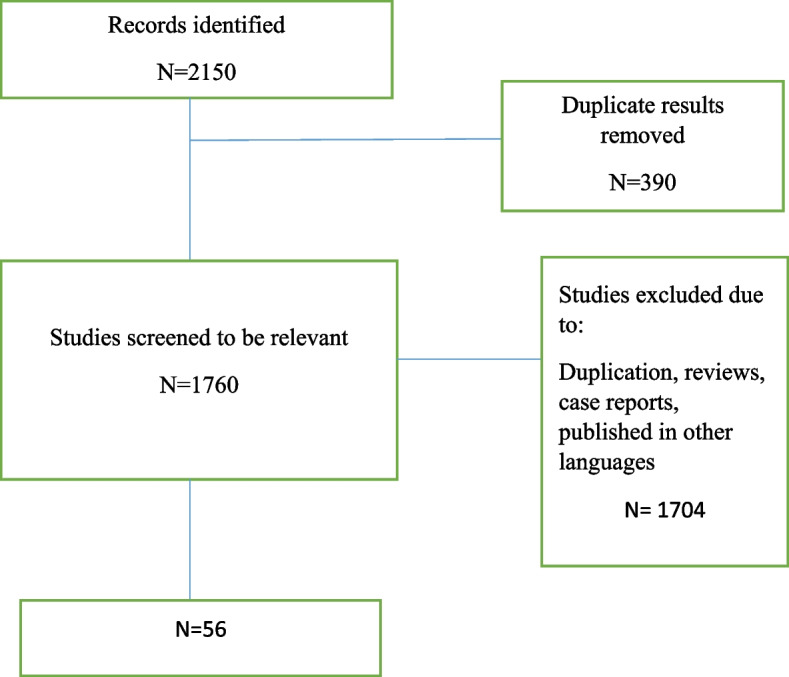
Flow diagram summarizing the selection of eligible studies

We found 2150 articles by literature search, after deleting duplicates 1760 remained. Fifty-six articles remained for meta-analysis (Fig. 1).

Included studies were published between 1996, and 2021.Most included studies were from Iran, followed by Italy, and Turkey. The mean age of participants ranged between 24.7, and 50.7 years, and EDSS ranged between 1.7 and 5. The most frequent applied questionnaire was FSFI (Table 1).

**Table 1:** Basic characteristics of included studies

Author	Year	Country	Design	T. MS All female	MS type RRMS SPMS PPMS CIS	Age	EDSS	Disease duration	Measurements	Total sexual dysfunction in PwMS	Anorgasmia
M Zorzon [12]	1999	Italy	Case control	70	RR 50 PP 16 SP 4	40.2(10.9)	2.6(1.7)	10.7(8.5)	Szasz Sexual Functioning Scale	44	21
M. Zorzon [20]	2001	Italy	Cohort	64	NR	NR	NR	NR	Szasz Sexual Functioning Scale	24	24
Kisic-Tepavcevic Darija [21]	2015	Serbia	cohort	66	NR	NR	NR	NR	Szasz sexual functioning scale	45	8
DK Tepavcevic [5]	2008	Serbia	cross-sectional	78	RR 54 SP 22 PP 2	41.7(9.3)	4.6(1.6)	9.1(6.7)	Szasz sexual functioning scale	66	12
Marita P. McCabe [22]	1996	Australia	Cross-sectional	74	NR	NR	NR	NR	Szasz At least one problem	59	17
Iris Zavoreo [23]	2016	Croatia	Cross sectional	56	NR	NR	NR	NR	SSS	NR	NR
Aleksandra Kołtuniuk [24]	2020	Poland	Cross-sectional	101	RR 82 SP 14 PP 5	36.7(9.56)	NR	75.1(50.2) months	SSQ	**55**	NR
M. Lew-Starowicz [25]	2013	Poland	Cross-sectional	137	NR	50.7(7)	NR	16.4(8.6)	SFQ28	113	NR
MW Nortvedt [26]	2001	Norway	Cross-sectional	118	NR	NR	NR	NR	SF-36	67	NR
Sacco E [27]	2011	Italy	Cross-sectional	65	NR	NR	NR	NR	PISQ-12	22	24
R Vazirinejad [28]	2008	Iran	Cross-sectional	126	NR	NR	NR	NR	MSQOL-54	115	NR
Jing Wu [29]	2020	Australia	Cross-sectional	1591	NR	NR	NR	NR	MSQOL-54	NR	NR
Z. Tulek [30]	2011	Turkey	cross-sectional	70	NR	NR	NR	NR	MSQOL-54	NR	NR
MW Nortvedt [31]	2007	Norway	Cross-sectional	40	NR	NR	NR	NR	MSQoL-54	NR	NR
Claudia H. Marck [32]	2016	Australia	Cross-sectional	1663	NR	NR	NR	NR	MSQOL-54	925	NR
Effat Merghati-Khoei [33]	2013	Iran	Cross-sectional	132	NR	36.9(8.3)	NR	6.8(5.3)	MSISQ-19	115	NR
Vida Ghasemi [34]	2020	Iran	Cross-sectional	260	RR 212 SP 36 PP 12	37.83(7.34)	2.02 (1.52)	6.96(5.06)	MSISQ-19 44.19(16)	198 Primary SD 176 Secondary SD 158 Tertiary SD 126	NR
Dilaram Billur Çelik [13]	2013	Turkey	Cross-sectional	44	NR	NR	NR	NR	MSISQ-19	32 Primary SD 19 Seconder SD 25 Tertiary SD 13	NR
M. Demirkiran [35]	2006	Turkey	Cross-sectional	33	NR	NR	NR	NR	MSISQ-19	27	NR
Sarah Abdo [36]	2020	Egypt	Cross-sectional ABS	43	NR	24.71(3.55)	NR	NR	MSISQ-19	24 Primary SD 43 secondary SD 7 Tertiary SD 43	NR
Edgar Carnero Contentti [37]	2019	Argentina	Cross-sectional	137	RR 112 PP 9 SP 18	49.1(10.2)	NR	7.5(0.5)	MSISQ-19	119 Primary SD 99 Secondary SD 103 Tertiary SD 88	NR
Patrick Altmann [38]	2021	Italy	cross-sectional	53	NR	NR	NR	NR	MSISQ-19	25	NA
Fereshteh Ashtari [39]	2014	Iran	cross-sectional	271	NR	36.1(8) *n* = 173 33.6(7.9) *n* = 98	NR	78.4(53.5) *n* = 173 60.4(36.8) *n* = 98	MSISQ-19	173 Primary SD 142 secondary SD 102 tertiary SD 120	NA
Kowsar Qaderi [40]	2014	Iran	Cross-sectional	132	NR	36.9(8.3)	NR	NR	MSISQ-19	110	NR
Sabine Salhofer-Polanyi [41]	2016	Austria	Cross-sectional	42	NR	34(7)	Median 1.75	NR	MSISQ-19	15 Primary SD 28 Secondary SD 17 Tertiary SD 14	NR
Hanna Pašiü [42]	2019	Croatia	Cross-sectional	75	NR	NR	NR	NR	MSISQ-15	NR	NR
Stenager E [43]	1996	Denmark	cohort	27	NR	NR	NR	NR	MRD	16	NR
Marita P. McCabe [44]	2002	Australia	Case–control	237	NR	44.45	NR	NR	ISS	194	NR
Marita P. Mccabe [45]	2003	Australia	Cohort	321	NR	NR	NR	NR	ISS	278	NR
Cira Fraser [14]	2008	USA	cross-sectional	219	NR	45.4(9.3)	NR	NR	Guy’s Neurological Disability Scale	106	NR
Simon Dupont [46]	1996	UK	Cross-sectional	65	NR	NR	NR	NR	GRISS	11	18
Vassilios Tzortzis [7]	2008	Greece	Cross-sectional	63	RR 58 PP 5	33(6.4)	mean 2.5, range 0–3.5	Mean 2.7 Range 19–51	FSFI	22	NR
Aleksandra Kołtuniuk [24]	2020	Poland	Cross-sectional	101	RR 82 SP 14 PP 5	36.7(9.56)	NR	75.1(50.2) months	FSFI	**45**	NR
Katharina M. Hösl [47]	2018	USA	Cross-sectional	83	RR 76 SP 6 PP 1	Median 36.2	NR	NR	FSFI	37	NR
Fatemeh Nazari [48]	2020	Iran	Cross-sectional	300	RR 243 PMS 39 CIS 18	36.35(7.33)	2.06(1.85)	7.37(5.40)	FSFI	209	NR
Pawel Bartnik [49]	2017	Poland	Cross-sectional	86	RR 86	32.03(7.22)	2.03(1.44)	7.87(5.38)	FSFI	21	NR
Marcin Popek [50]	2018	Poland	case–control	55	NR	NR	NR	NR	FSFI 26.24(7.22)	22	NR
Fatih Firdolas [51]	2012	Turkey	Cross sectional	23	RR 17 SP 6	NR	2(0.22) *N* = 17 5.91(0.53) *N* = 6	NR	FSFI	12	NR
Giulia Gava [52]	2019	Italy	Case–control	153	NR	47.3(10.5)	3.1(2.2)	13.5(8.7)	FSFI 17.9(12.7)	64	NR
Ilan Gruenwald [53]	2007	Israel	cross-sectional	41	RR 38 SP 3		Median 2.5	Median 10	FSFI	25	NR
Charalampos Konstantinidis [54]	2018	Greece	cross-sectional	248	NR	45.84(8.448)	NR	12.78(2.18)	FSFI	160	NR
Giuseppe Lombardi [55]	2011	Italy	Cross-sectional	54	NR	Mean:34.7 (26–44)	Mean:2.9 (1.5–6)	Mean 8.6 (2–18)	FSFI	31	NR
Fariba Askari [2]	2016	Iran	Cross-sectional	86	RR 81 SP 5	33.4(6.5)	NR	NR	FSFI	58	NR
Khadijeh Mohammadi [56]	2013	Iran	cross-sectional	226	RR 169 PP 4 SP 53	35.7(8.07)	NR	1.8(0.79)	FSFI	125	NR
Alireza Alehashemi [57]	2019	Iran	case–control	64	RR 60 SP 4	35.25(8.07)	Mean 2 Range 0–6	Mean 52.5 months (ranging from 6 to 84.5)	FSFI 22.86 (5.36)	53	NR
Ramezani, M.A [58]	2018	Iran	Cross-sectional	70	NR	NR	NR	NR	FSFI	44	NR
Jeroen R. Scheepe [59]	2015	Netherlands	Cross-sectional	50	NR	NR	NR	NR	FSFI	16	NR
Tzitzika, M [54]	2018	Greece	Cross-sectional ABS	267	NR	NR	NR	NR	FSFI	172	NR
Julia Koehn [60]	2014	Germany	Cross-sectional	82	NR	36.7(9.5)	NR	69(75.1) Months	FSFI 3.31(1.2)	37	NR
Mahsa Ghajarzadeh [11]	2013	Iran	Case–control	100	RR 95 SP 5	32.8(7.6)	5(4.8)	13(3.1)	FSFI 23.2(7.1)	66	NR
Volkan Solmaz, [61]	2018	Turkey	Case–control	42	RR 34 SP 7 PP 1	41.9(8.06)	Median 2.2(0–7)	Mean range: 8.9(25–1)	FSFI 15.84(6.33)	40	NR
E. Fragala [62]	2015	Italy	Cross-sectional	75	NR	NR	NR	NR	FSFI median (IQR) 16.0 (2.0–25.5)	51	NR
Marian Petersen [63]	2020	Denmark	Cross-sectional	180	NR	NR	NR	NR	CSFQ	116	NR
Dilaram Billur Çelik [13]	2013	Turkey	Cross-sectional	44	NR	NR	NR	NR	ASEX	NR	NA
Betu¨l Kılıc [64]	2012	Turkey	cross-sectional	23	NR	Mean 39.83 (8.88)	NR	8.09(7.29)	Arizona Sexual Experiences Scale 17.91(5.75)	14	NR
Rocco Salvatore CalabrJ [65]	2018	Italy	Cross-sectional	54	NR	NR	NR	NR	40-item ad hoc questionnaire	NR	31

Author	Hyporgasmia	Decrease vaginal lubrication	Change in vaginal sensation	Reduce libido	Painful intercourse	Lack of sexual desire	Partner satisfaction problems	satisfaction	Arousal Problems	T control	Total sexual dysfunction in control
M Zorzon [12]	17	25	19	22	NR	NR	NR	NR	NR	71	8
M. Zorzon [20]		25	25	28	NR	NR	NR	NR	NR	NA	NA
Kisic-Tepavcevic Darija [21]	27	27	18	47	NR	NR	NR	NR	NR	NA	NA
DK Tepavcevic [5]	35	30	21	58	NR	NR	NR	NR	NR	NA	NA
Marita P. McCabe [22]	NR	14	NR	NR	1	21	NR	NR	NR	NA	NA
Iris Zavoreo [23]	12	11	NR	NR	NR	14	NR	NR	NR	NA	NA
Aleksandra Kołtuniuk [24]	NR	NR	NR	NR	NR	NR	NR	NR	NR		
M. Lew-Starowicz [25]	54	66	65	NR	NR	79	NR	NR	62	NA	NA
MW Nortvedt [26]	NR	NR	NR	NR	NR	NR	NR	NR	NR	NA	NA
Sacco E [27]	NR	NR	NR	NR	20	21	NR	29	NR	NA	NA
R Vazirinejad [28]	NR	NR	NR	NR	NR	NR	NR	NR	NR	NA	NA
Jing Wu [29]	929	808	NR	NR	NR	1017	549	NR	NR	NA	NA
Z. Tulek [30]	35	28	NR	NR	NR	37	23	NR	NR	NA	NA
MW Nortvedt [31]	28	20	NR	NR	NR	25	18	NR	NR	NA	NA
Claudia H. Marck [32]	599	514	NR	NR	NR	695	238	NR	NR	NA	NA
Effat Merghati-Khoei [33]	100	80	46	77	NR	NR	NR	NR	NR	NA	NA
Vida Ghasemi [34]	156	82	31	NA	NR	966	NR	NR	NR	NA	NA
Dilaram Billur Çelik [13]	10	10	NR	NR	NR	9	NR	NR	NR	NA	NA
M. Demirkiran [35]	22	17	19	21	NR	NR	NR	NR	20	NA	NA
Sarah Abdo [36]	NR	3	NA	11	NR	NR	NR	NR	NR	NA	NA
Edgar Carnero Contentti [37]	55	44	27	NR	NR	53	NR	NA	NA	NA	NA
Patrick Altmann [38]	NA	NA	NA	NA	NA	NA	NA	NA	NA	NA	NA
Fereshteh Ashtari [39]	112	70	48	NR	NR	92	NR	NR	NR	NA	NA
Kowsar Qaderi [40]	83	67	38	64	NR	NR	NR	NR	NR	NA	NA
Sabine Salhofer-Polanyi [41]	17	12	6	20	NR	NR	NR	NR	11	NA	NA
Hanna Pašiü [42]	19	16	13	NR	NR	19	11	NR	NR	NA	NA
Stenager E [43]	3	4	6	15	NR	NR	NR	NR	NR	NA	NA
Marita P. McCabe [44]	101	78	81	NR	40	96	NR	NR	73	190 mean age = 44.35 years	146
Marita P. Mccabe [45]	101	78	81	NR	40	96	NR	NR	73	239	191
Cira Fraser [14]	NR	NR	5	NR	NR	15		2		NA	NA
Simon Dupont [46]	NR	NR	NR	NR	26	NR	NR	7	NR	NA	NA
Vassilios Tzortzis [7]	NR	NR	NR	NR	NR	NR	NR	NR	NR	61	13
Aleksandra Kołtuniuk [24]	NR	NR	NR	NR	NR	NR	NR	NR	NR	NA	NA
Katharina M. Hösl [47]	NR	NR	NR	NR	NR	NR	NR	NR	NR	21	1
Fatemeh Nazari [48]	111	71	NR	NR	51	116	NR	70	116	NA	NA
Pawel Bartnik [49]	NR	NR	NR	NR	NR	NR	NR	NR	NR	NA	NA
Marcin Popek [50]	NR	NR	NR	NR	NR	NR	NR	NR	NR	55 29.91 (3.79)	12
Fatih Firdolas [51]	NR	NR	NR	NR	NR	NR	NR	NR	NR	NA	NA
Giulia Gava [52]	NR	NR	NR	NR	NR	NR	NR	NR	NR	153 Age:48.5(9.6) FSFI:21.1(11.2)	24
Ilan Gruenwald [53]	22	NR	NR	NR	3	25	NR	NR	13	NA	NA
Charalampos Konstantinidis [54]	NR	NR	NR	NR	NR	NR	NR	NR	NR	NA	NA
Giuseppe Lombardi [55]	NR	NR	NR	NR	NR	12	NR	NR	NR	NA	NA
Fariba Askari [2]	NR	NR	NR	NR	NR	NR	NR	NR	NR	NA	NA
Khadijeh Mohammadi [56]	81	41			29	77		54	100	NA	NA
Alireza Alehashemi [57]	NR	NR	NR	NR	NR	NR	NR	NR	NR	**64** **24.39 (4.75)**	NR
Ramezani, M.A [58]	NR	NR	NR	NR	36	21	NR	NR	NR	NA	NA
Jeroen R. Scheepe [59]	NR	NR	NR	NR	NR	NR	NR	NR	NR	NA	NA
Tzitzika, M [54]	NR	NR	NR	NR	NR	NR	NR	NR	NR	NA	NA
Julia Koehn [60]	NR	NR	NR	NR	NR	NR	NR	NR	NR	21 Age 36.5(11.8) FSFI: 4.5(0.7)	1
Mahsa Ghajarzadeh [11]	NR	NR	NR	NR	NR	NR	NR	NR	NR	50 Age 31.8(8.4) FSFI: 26.8(5.2)	NR
Volkan Solmaz, [61]	NR	NR	NR	NR	NR	NR	NR	NR	NR	41 Age:39.7(7.3) FSFI:31.01(3.53)	NR
E. Fragala [62]	NR	NR	NR	NR	NR	NR	NR	NR	NR	NA	NA
Marian Petersen [63]	118	NR	NR	NR	NR	131	NR	131	151	NA	NA
Dilaram Billur Çelik [13]	4	5	NA	5	NR	NR	NR	6	7	NA	NA
Betu¨l Kılıc [64]	NR	NR	NR	NR	NR	NR	NR	NR	NR	NA	NA
Rocco Salvatore CalabrJ [65]	NR	NR	NR	NR	NR	NR	NR	NR	NR	NA	NA

Totally 8980 patients were evaluated and the total number of patients with SD was 4245.

**Fig. 2:**
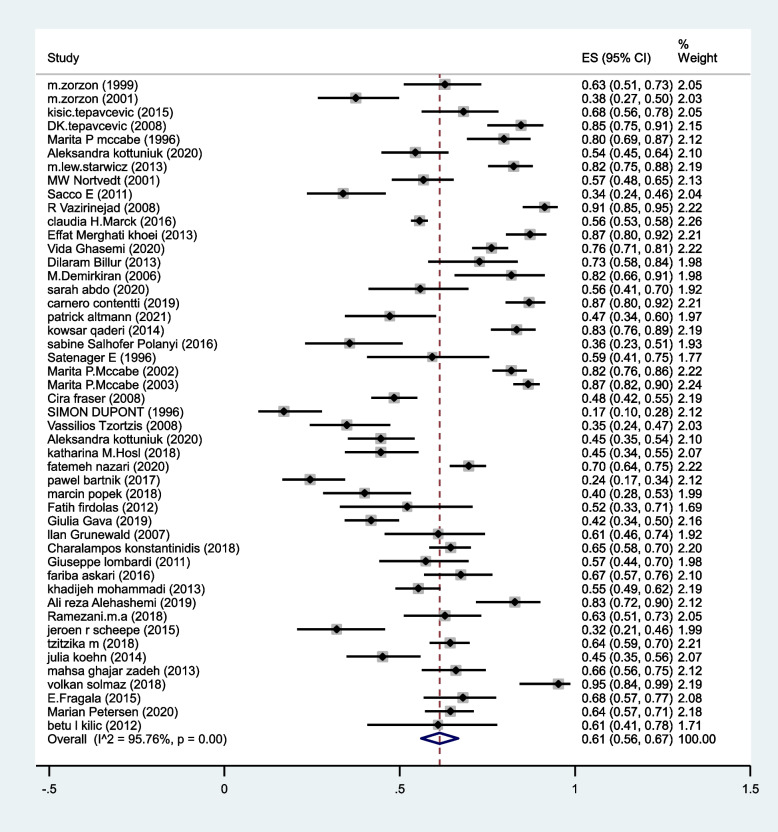
The pooled prevalence of SD in MS patients

The pooled prevalence of SD in MS patients estimated as 61% (95%CI:56–67%) (I^2^:95.7%, *P* < 0.001) (Fig. 2).

**Fig. 3:**
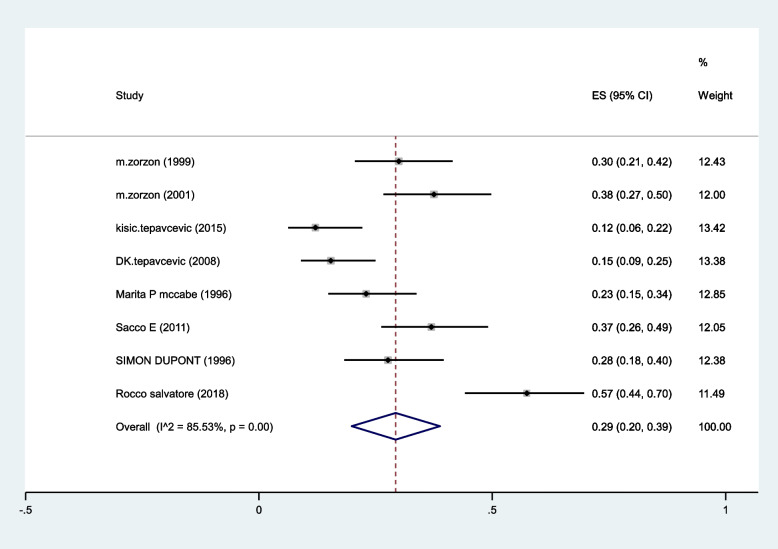
The pooled prevalence of anorgasmia in MS patients

The pooled prevalence of Anorgasmia in MS patients estimated as 29% (95%CI:20–39%) (I^2^:85.3%, *P* < 0.001) (Fig. 3).

**Fig. 4:**
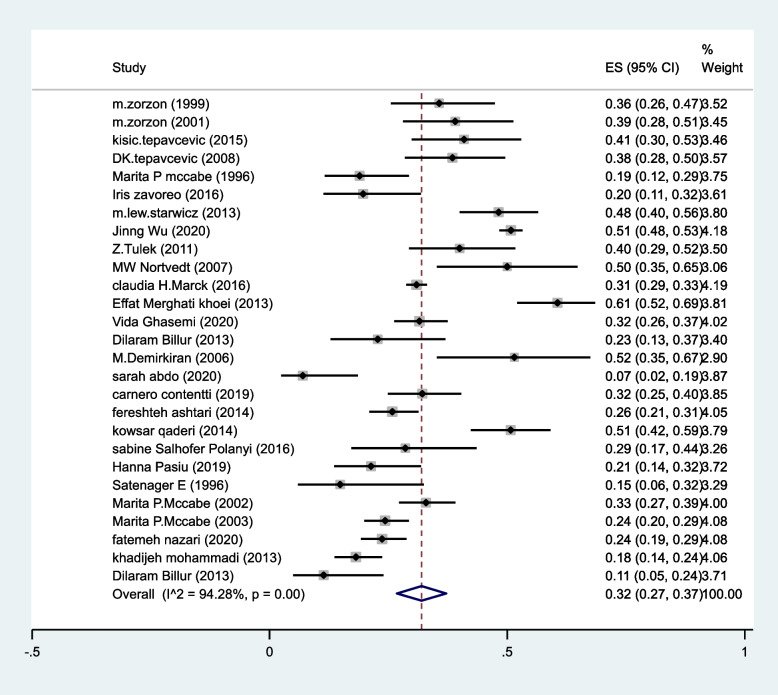
The pooled prevalence of decreased vaginal lubrication in MS patients

The pooled prevalence of decreased vaginal lubrication in MS patients estimated as 32%(95%CI:27–37%) (I^2^ = 94.2%, *P* < 0.001) (Fig. 4).

**Fig. 5:**
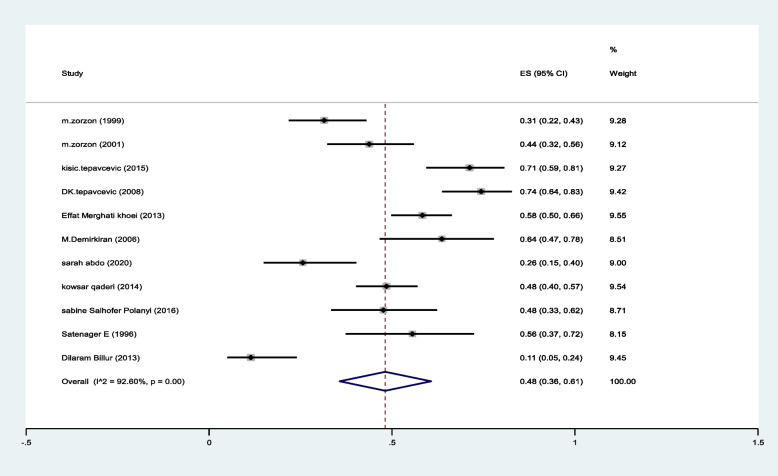
The pooled prevalence of reduced libido in MS patients

The pooled prevalence of reduced libido was 48%(95%CI:36–61%) (I^2^:92.6%, *P* < 0.001) (Fig. 5).

**Fig. 6:**
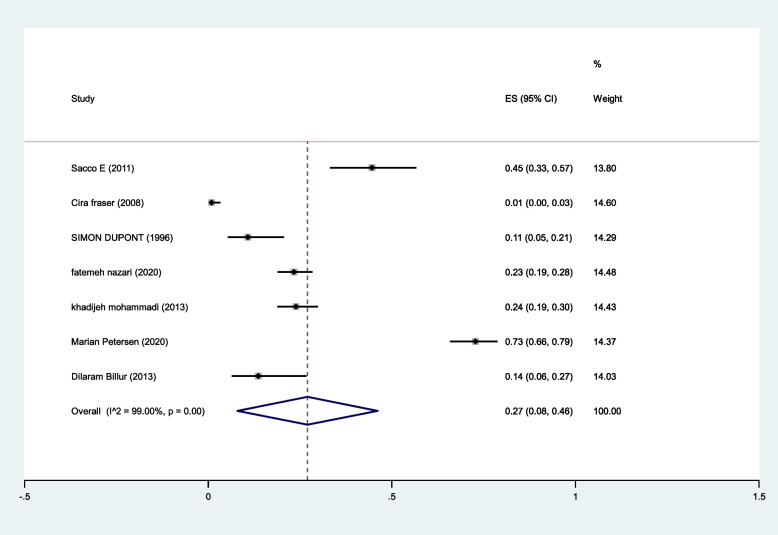
The pooled prevalence of reduced libido in MS patients

The pooled prevalence of intercourse satisfaction was 27% (95%CI: 8–46%) (I^2^:99%, *P* < 0.001) (Fig. 6).

**Fig. 7:**
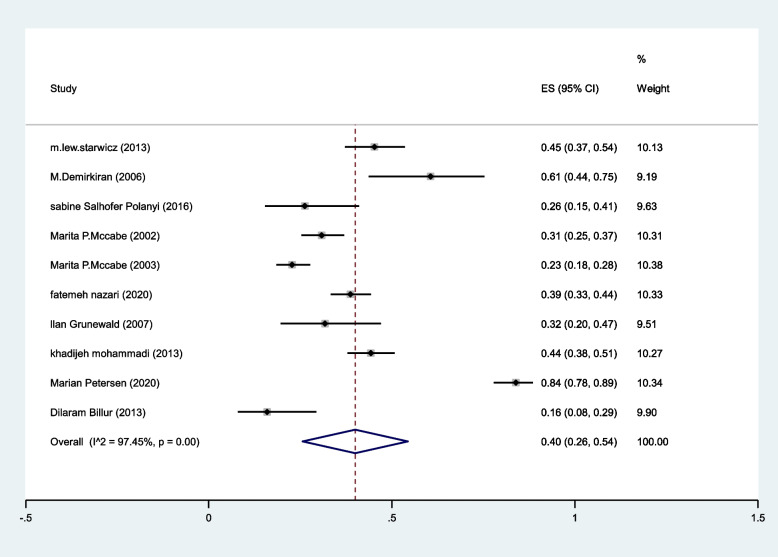
The pooled prevalence of arousal problems in MS patients

The pooled prevalence of arousal problems was 40%(95%CI: 26–54%) (I^2^:97.4%, *P* < 0.001) (Fig. 7).

**Fig. 8:**
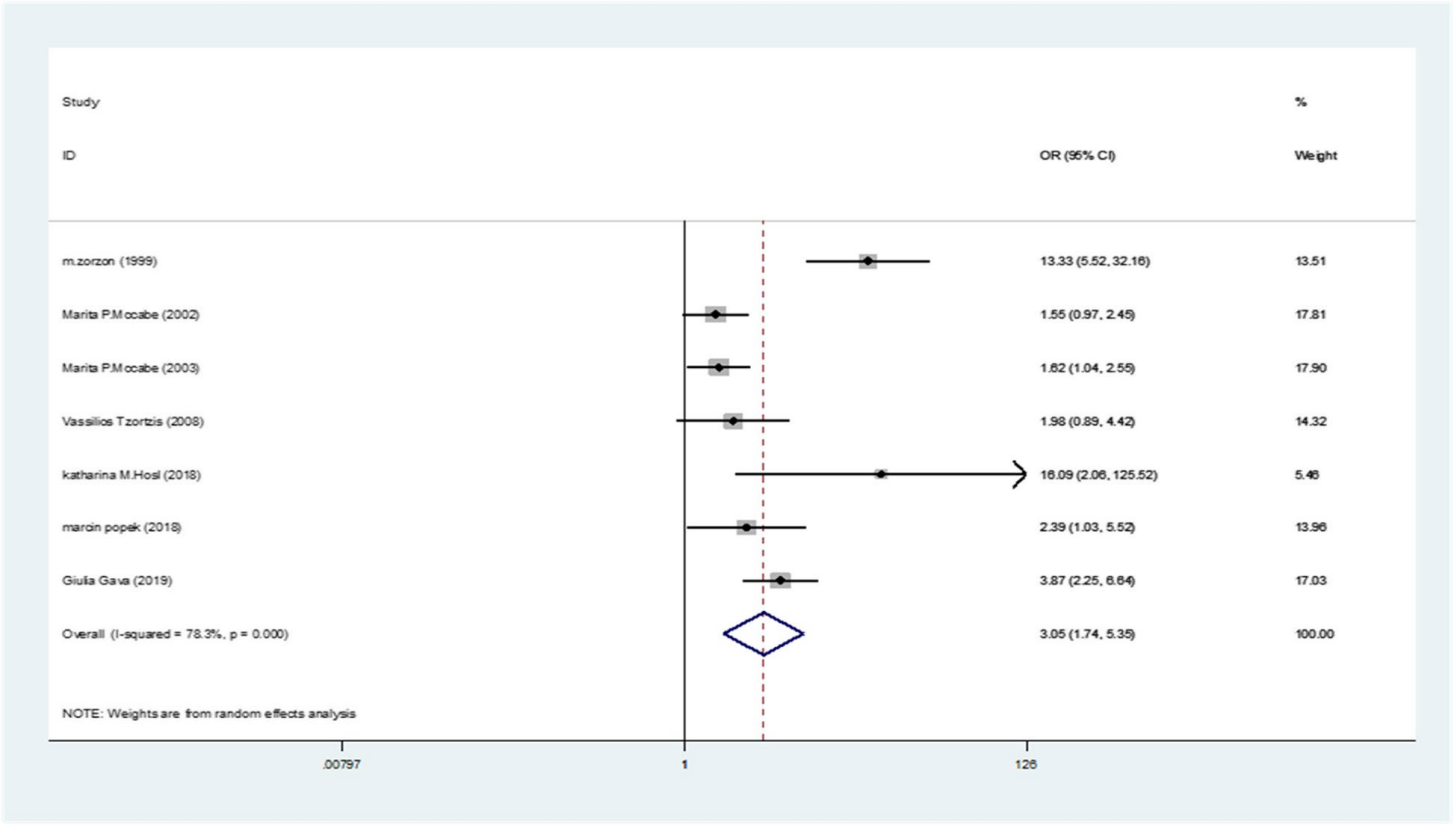
The pooled odds of developing SD in women with compared to healthy controls

The pooled odds of developing SD in MS women estimated as 3.05(95%CI: 1.74–5.35) (I^2^:78.3%, *P* < 0.001) (Fig. 8).

## Discussion

MS is a neurological disease characterized by a wide range of physical, and psychological complications. The prevalence of SD in MS is near five times higher than general population [12, 66], although it is considered poorly in this population.

To our knowledge, this is the first comprehensive systematic review and meta-analysis including all related studies evaluating SD in women with MS. We included all studies which used different questionnaires. The pooled prevalence of SD was estimated as 61%, and the most common SD complaint was reduced libido (the pooled prevalence was estimated as 48%), we also found that the pooled prevalence of intercourse satisfaction was 27%.

We included all studies which applied different diagnostic tools, so out estimate would be higher than previous ones.

In a previous systematic review and meta-analysis which was conducted by Zhao et al., the relative risk (RR) of developing SD in MS women was 1.87 which shows that women with MS have 87% increased risk of developing SD [16]. They also reported lower pooled scores of desires, arousal, orgasm, satisfaction, pain, and lubrication in MS group.

In our previous systematic review, which we included only studies that applied FSFI questionnaire for evaluating SD in MS, the pooled prevalence of SD estimated as 55% [18].

In 2008, Tzorts et al. evaluated 63 women with MS using FSFI questionnaire, and reported SD in22 and reported no depression in affected cases [7].

Zorzon et al. used Szasz Sexual Functioning Scale for SD assessment and reported SD in 44 out of 70 cases. Anorgasmia or hyporgasmia followed by decreased vaginal lubrication were the most affected subscales [12].

The variation about the prevalence of SD in included studies is due to unclear definition of SD, diverse inclusion, and exclusion criteria, various diagnostic methods, no standardized tools, and cultural issues.

SD is an important issue in marital life which is ignored by most physicians and patients. It is a multi-dimensional issue which affects quality of life as well as psychological well-being. Different factors such as disease duration, disability level, psychological disorders such as depression, anxiety, and stress are considered to play role in SD development in MS while there is controversies between studies [11].

Most physicians do not pay attention to this part of their patient’s lives, and patients hesitate to talk about intimate issues.

Depression is negatively correlated with FSFI score and its subscales in a previous original study [11]. On the other hand, it is shown that depression is related with both libido reduction and arousal problems [67, 68].

In another study, higher age was associated with increased SD prevalence in MS [69] while Çelik et al. reported that SD should be evaluated in MS women at earlier stages and disease duration or disability level are not prognostic factors for developing SD [13]. Zhao et al. in their meta-analysis showed that disease duration longer than ten years, increases the risk of SD 2.5fold in MS cases [16].

Another influencing factor is bladder dysfunction in MS cases which negatively affects their sexual activity [70]. Fragala et al. investigated SD in 91% of MS women with detrusor over-activity and 66% without detrusor over-activity [62].

The association refers to S2, S3 and S4 innervation of bladder, which control sexual response [10]. On the other hand, detrusor dysfunction as a MS-related complication may reflect severe neurological disability and SD [71].

Zivadinov et al. investigated that physical disorders, depression, age at MS onset, and also neurological impairment while they reported no correlation between SD and duration of the disease [15].

Higher disability level, depression and anxiety were related with SD presence in Demirkian et al. study [72].

This systematic review has some strength. First, we included all studies which evaluated SD. Second, the number of included studies is high. Third, we analyzed all subscales of SD.

We also had some limitations. First, all included studies used various diagnostic tools. Second, there was no clear definition of SD. Third, inclusion criteria of participants differed between studies.

## Conclusion

The result of this systematic review and meta-analysis show that the pooled prevalence of SD in women with MS is 61% and the odds of developing SD in comparison with controls is 3.05.

## Data Availability

All data generated or analyzed during this study are included in this published article.
